# Clinical and imaging features of myeloid sarcoma: a German multicenter study

**DOI:** 10.1186/s12885-019-6357-y

**Published:** 2019-11-27

**Authors:** Hans-Jonas Meyer, Wolfram Pönisch, Stefan Andreas Schmidt, Susanne Wienbeck, Friederike Braulke, Dominik Schramm, Alexey Surov

**Affiliations:** 1 Department of Diagnostic and Interventional Radiology, University of Leipzig, University Hospital Leipzig, Liebigstraße 20, 04103 Leipzig, Germany; 2 Department of Hematology and Oncology, University Hospital Leipzig, Leipzig, Germany; 3 Department of Diagnostic and Interventional Radiology, Ulm University Medical Center, 89081 Ulm, Germany; 4 Department of Diagnostic and Interventional Radiology, University Medicine Göttingen, 37075 Göttingen, Germany; 5 Department of Hematology and Medical Oncology, University Medicine Göttingen, 37075 Göttingen, Germany; 6 Department of Diagnostic and Interventional Radiology, University Hospital of Halle (Saale), 06097 Halle (Saale), Germany

**Keywords:** Myeloid sarcoma, Granulocytic sarcoma, Chloroma, Acute myeloid leukemia

## Abstract

**Background:**

Myeloid sarcoma (MS), also known as chloroma, is an extramedullary manifestation of malignant primitive myeloid cells. Previously, only small studies investigated clinical and imaging features of MS. The purpose of this study was to elucidate clinical and imaging features of MS based upon a multicenter patient sample.

**Methods:**

Patient records of radiological databases of 4 German university hospitals were retrospectively screened for MS in the time period 01/2001 and 06/2019. Overall, 151 cases/76 females (50.3%) with a mean age of 55.5 ± 15.1 years and 183 histopathological confirmation or clinically suspicious lesions of MS were included into this study. The underlying hematological disease, localizations, and clinical symptoms as well as imaging features on CT and MRI were investigated.

**Results:**

In 15 patients (9.9% of all 151 cases) the manifestation of MS preceded the systemic hematological disease. In 43 cases (28.4%), first presentation of MS occurred simultaneously with the initial diagnosis of leukemia, and 92 (60.9%) patients presented MS after the initial diagnosis. In 37 patients (24.5%), the diagnosis was made incidentally by imaging. Clinically, cutaneous lesions were detected in 35 of 151 cases (23.2%). Other leading symptoms were pain (*n* = 28/151, 18.5%), neurological deficit (*n* = 27/151, 17.9%), swelling (*n* = 14/151, 9.3%) and dysfunction of the affected organ (*n* = 10/151, 6.0%). Most commonly, skin was affected (*n* = 30/151, 16.6%), followed by bone (*n* = 29/151, 16.0%) and lymphatic tissue (*n* = 21/151, 11.4%). Other localizations were rare. On CT, most lesions were homogenous. On T2-weighted imaging, most of the lesions were hyperintense. On T1-weighted images, MS was hypointense in *n* = 22/54 (40.7%) and isointense in *n* = 30/54 (55.6%). A diffusion restriction was identified in most cases with a mean ADC value of 0.76 ± 0.19 × 10^− 3^ mm^2^/s.

**Conclusions:**

The present study shows clinical and imaging features of MS based upon a large patient sample in a multicenter design. MS occurs in most cases meta-chronous to the hematological disease and most commonly affects the cutis. One fourth of cases were identified incidentally on imaging, which needs awareness of the radiologists for possible diagnosis of MS.

## Background

Myeloid sarcoma (MS), also known as granulocytic sarcoma or chloroma, is a solid extramedullary tumor originating from malignant primitive myeloid cells [1–3]. Its occurrence is linked to leukemic diseases of the myeloid cell line, most commonly in acute myeloid leukemia (AML) and less commonly in chronic myeloid leukemia (CML), myelodysplastic syndrome (MDS) or other myeloproliferative disorders [4].

Most frequently, it is a recurrence of the primary disease, less commonly concurrently with the primary disease or it can even be the initial manifestation of the hematological disease, which can impose great diagnostic challenges [4, 5]. A very rare form is the primary extramedullary manifestation of AML with normal appearing bone marrow, which comprises less than 1% of all patients with AML [6].

An identified risk factor for MS is allogenic bone marrow transplantation [7]. The overall survival of patients with MS is poor with a median survival of 12.8 months [6].

MS can possibly occur in any organ system, which leads to a great amount of differential diagnosis, including other benign and malignant tumors, hematomas and inflammatory diseases depending on the localization [2, 4, 8].

Currently, the body of literature investigating radiological findings of MS is composed of small patient samples based upon single center studies with inherent limitations [9–11].

Therefore, the purpose of this study was to evaluate clinical and imaging characteristics of MS based upon a large patient sample in a multicenter design.

## Methods

### Patient sample

The institutional ethic committee waived the need for informed consent due to the retrospective nature of the study (Committee of the University of Leipzig, Study codes Nr. 027/2002 and 162/2004). No permission was required to review the patient records. The radiology databases of 4 German university hospitals (University of Leipzig, Martin-Luther University of Halle (Saale), University Medicine of Göttingen, Ulm University Medical Center) were retrospectively screened for myeloid sarcoma in the time period between 01/2001 and 06/2019. Cases were included into the study either with histopathological confirmation of MS (*n* = 109/151, 72.2% of all included patients) or with clinical highly suspicious lesions of MS with histopathological confirmed of associated hematological disease (*n* = 42/151, 27.8% of all included patients). The overall patient sample was comprised of 151 patients with a median age of 55.5 ± 15.1 years (range 16–86 years). There were 76/151 female patients (50.3%) and 75/151 (49.7%) male patients.

### Clinical features

The patient records were center specific reviewed. The underlying hematological disease was categorized in following subgroups: AML, CML, myeloproliferative syndrome, not further classified (MPS), MDS, primary idiopathic myelofibrosis, and chronic myelomonocytic leukemia (CMML). The kind of first clinical manifestation was evaluated and sorted as followed: neurological deficit; incidental finding by imaging; pain; skin-related color changes or swelling; organ-specific dysfunction.

### Image analysis

For 57/151 patients (37.7%) computed tomography (CT) images and for 54/151 patients (35.8%) magnetic resonance imaging (MRI) were available.

Different CT scanners (Somatom Sensation 64, Somatom Definition AS 128 Siemens, Erlangen, Germany; Ingenuity and Brilliance iCT 256, Philips Medical Systems, Cleveland, OH, USA) were used. CT scans included cervical, thoracic, abdominal, and pelvic regions. In all patients 1.5 ml of iodinated intravenous contrast medium per body mass were given at a rate of 1.5–2.5 ml/s by a power injector, with a scan delay of approximately 90 s (portal venous phase) after onset of injection.

For 2/151 patients (1.3%) F^18^-fluorodexyglucose-(FDG-PET)-Positron-emission tomography (Siemens Biograph 16, Siemens Medical Solutions, Erlangen, Germany) was available.

For MRI, the following scanners were used: 3.0 T Magnetom Trio, 1.5 T Magnetom Vision Sonata Upgrade, 1.5 T Magnetom Aera, Magnetom Skyra 3.0 T, Magnetom Avanto 1.5 T (Siemens Medical Solutions, Erlangen, Germany).

All imaging studies were performed in clinical routine with routine protocols.

The imaging studies were re-evaluated by the board-certified radiologists of every study center in awareness of the diagnosis. Following features were evaluated: type of modality (CT, MRI), maximum size (largest diameter in mm, defined on the slide with the largest tumor appearance), number of lesions, localization, imaging appearance (hypodense/hypointense, isodense/isointense, hyperdense/hyperintense in comparison to surrounding muscle tissue) and type of enhancement (no enhancement; homogenous or heterogeneous enhancement).

### Statistical analysis

Collected data were analyzed by means of descriptive statistics (absolute and relative frequencies) with SPSS (SPSS 25.0, SPSS Inc., Chicago IL, USA). Continuous variables were expressed as means ± standard deviation (SD), and categorical variables as percentages. Mann-Whitney test was used for group comparisons.

## Results

### Clinical findings

Overall, 95/151 MS patients (62.9%) suffered from primary AML, 29/151 (19.2%) from secondary AML, (26 from MDS and 3 MPS), 18/151 (11.9%) from CML, 3/151 (2.0%) from MDS, 3/151 (2.0%) from CMML, 2/151 (1.3%) from MPS, and 1/151 case (0.7%) from primary idiopathic myelofibrosis.

In 15/151 cases (9.9%), the manifestation of MS preceded the systemic hematological disease. In 43/151 cases (28.4%) first presentation of MS occurred simultaneously with the initial diagnosis of leukemia, and 92/151 (60.9%) patients presented MS after the initial diagnosis.

In 37/151 patients (24.5%), the lesions were detected incidentally by imaging. Clinically, typical cutaneous lesions were detected in 35/151 cases (23.2%). Other leading symptoms were pain (*n* = 28/151, 18.5%), neurological deficit (*n* = 27/151, 17.9%), swelling (*n* = 14/151, 9.3%) and dysfunction of the affected organ (*n* = 10/151, 6.0%).

### Localizations of MS

In 128/151 patients (84.8%), MS was limited to one localization. Two different localizations occurred in 17/151 patients (11.3%), three in 3/151 patients (2.0%) and four localizations in 3/151 patients (2.0%).

Most commonly, cutis was affected (*n* = 30/151, 16.6%), followed by bone (*n* = 29/151, 16.0%) and lymphatic tissue (*n* = 21/151, 11.4%). Other localizations were rare (Table 1).

**Table 1:** The identified localizations of Myeloid sarcoma. Twenty-three patients had 2 or more localizations resulting in overall 183 different lesions of Myeloid sarcoma in 26 different localizations

Localization	Lesion number (N)	Percent (%)
Skin	30	16.6
Bone	29	16.0
Lymphatic tissue	21	11.4
Breast	17	9.3
Central nervous system (brain)	17	9.3
Muscle	13	7.2
Central nervous system (spine)	8	4.4
Orbita	5	2.7
Central nervous system (meningeal)	3	1.6
Testis	3	1.6
Paranasal sinus	3	1.6
Vagina	3	1.6
Larynx	3	1.6
Kidney	2	1.1
Pancreas	2	1.1
Pleural	2	1.1
Salivary gland	2	1.1
Stomach	2	1.1
Cardiac	2	1.1
Uterus	2	1.1
Pharynx	2	1.1
Bladder	1	0.5
Peritoneal	1	0.5
Pulmonary	1	0.5
Ovarian	1	0.5
Liver	1	0.5
All	183	100

### Imaging findings

**Fig. 1:**
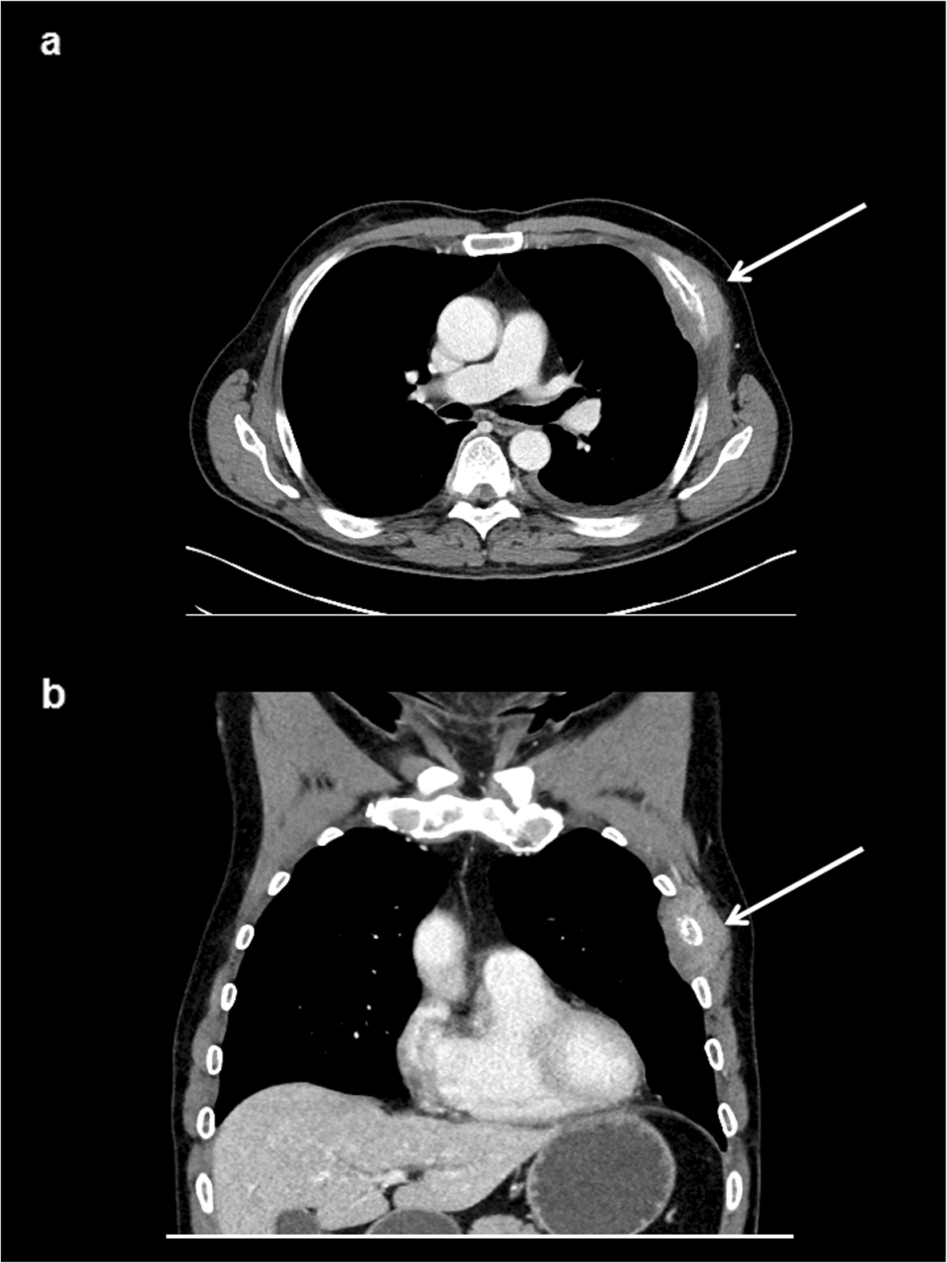
Myeloid sarcoma of the chest wall in a male patient with known acute myeloid leukemia in a relapse setting. Contrast enhanced computed tomography in axial (**a**) and coronal plane (**b**) showing a relatively homogenous of the third left rip with an infiltration of the adjacent intercostal muscles (arrows)

**Fig. 2:**
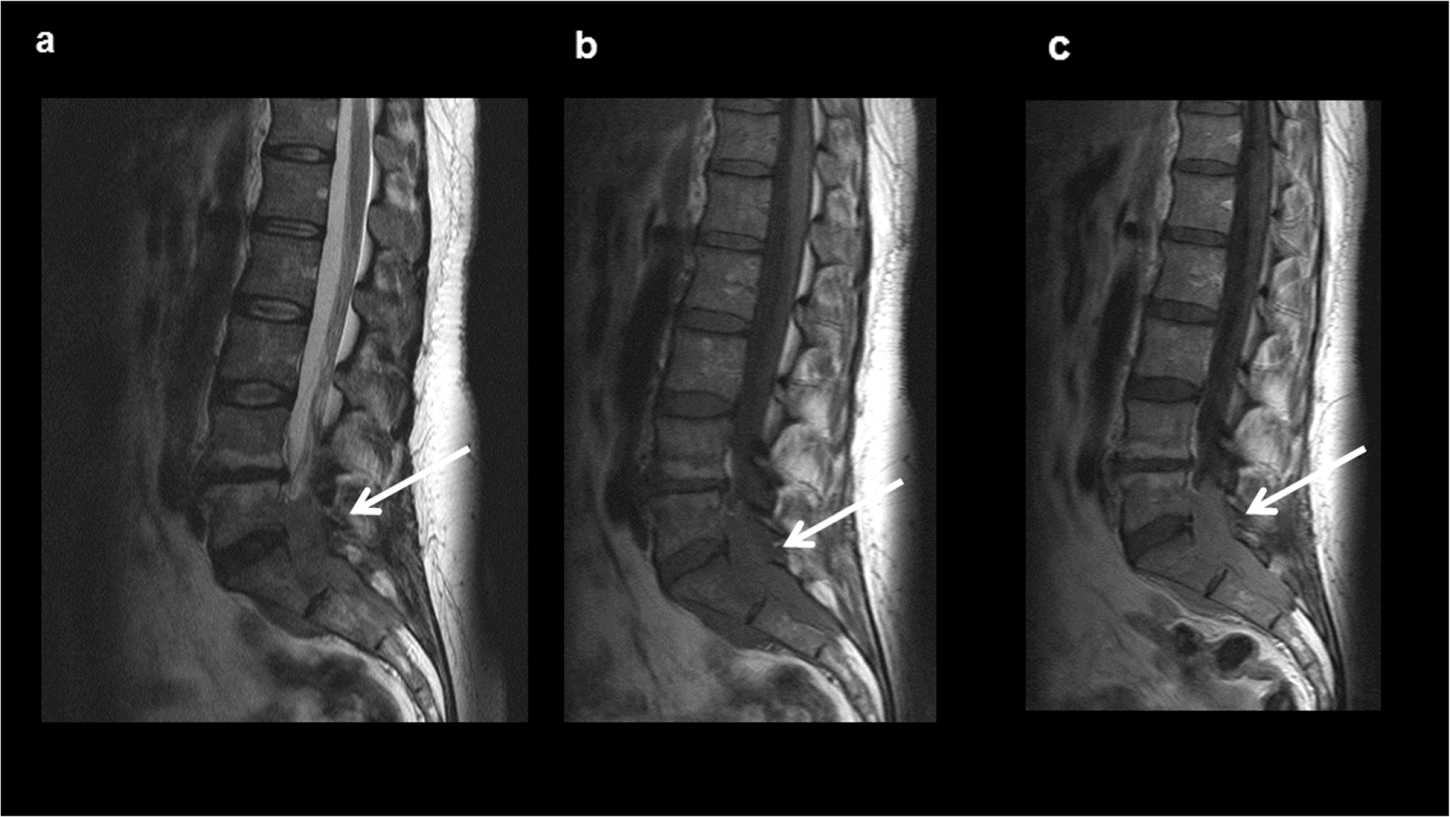
Intraspinal manifestation of Myeloid sarcoma in the lumbosacral region in a female patient with known acute myeloid leukemia in a relapse setting. **a** On sagittal T2-weighted image, the lesion is homogenous, hyperintense compared to adjacent muscle tissue (arrow). **b** On sagittal T1-weighted image, the lesion is hypointense (arrow). **c** On sagittal T1- weighted image after application of contrast medium a strong homogenous contrast enhancement can be appreciated (arrow)

**Fig. 3:**
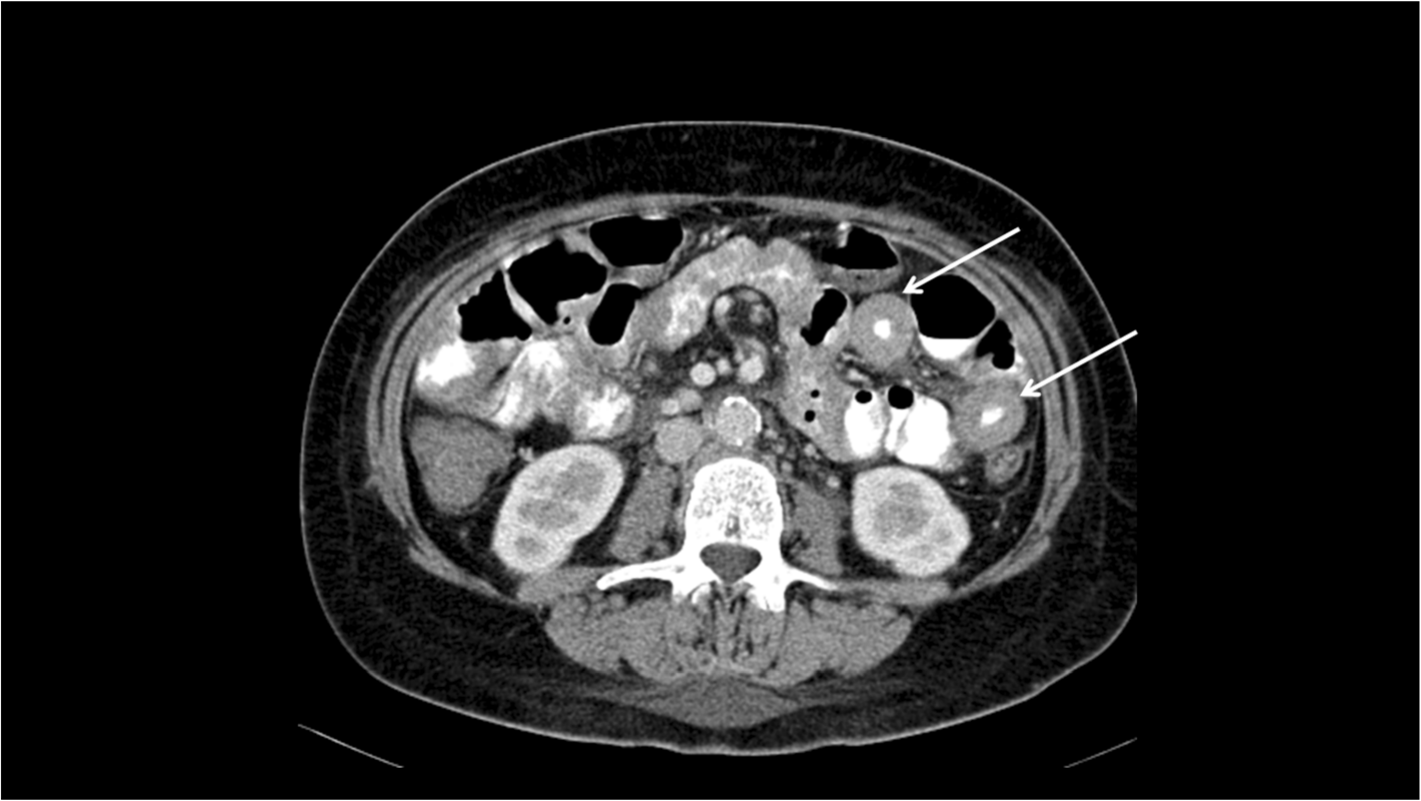
Small bowel affection of Myeloid sarcoma in a male patient with no known hematological disease. Acute myeloid leukemia was histologically diagnosed with biopsy of this lesion. Axial computed tomography showing multiple circular thickened jejunal loops. The diagnosis of Myeloid sarcoma was histopathologically confirmed after bowel biopsy

**Fig. 4:**
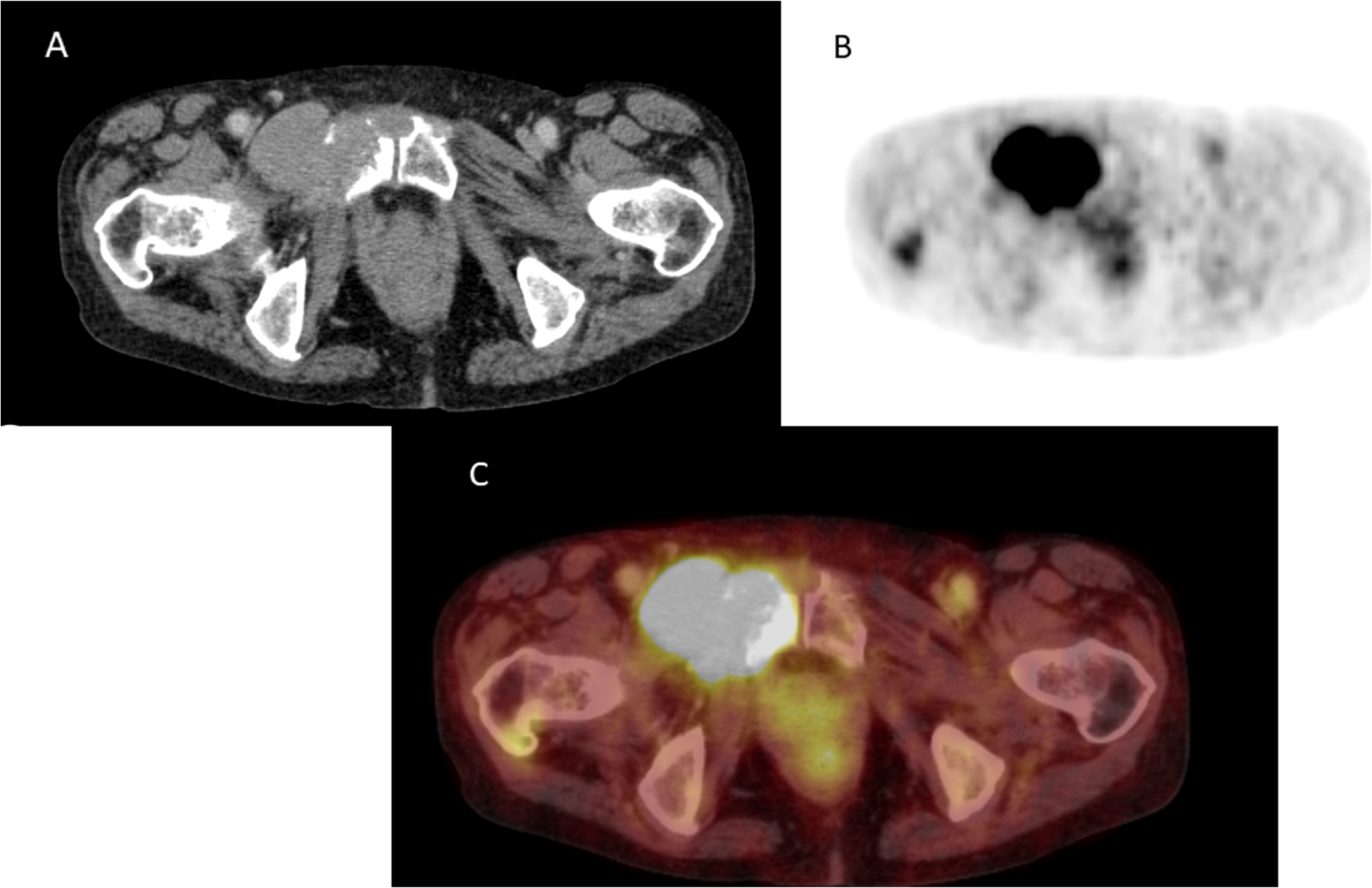
Myeloid sarcoma of the pelvis in a female patient with known acute myeloid leukemia. **a** Axial contrast media enhanced computed tomography shows a homogenous bone lesion of the right pubic bone with infiltration of the adjacent muscle. **b** On F18-fluorodexyglucose-Positron-emission tomography a high tracer uptake can be appreciated of the lesion. **c** Corresponding fused F18-fluorodexyglucose-Positron-emission tomography

Figures 1, 2, 3 and 4 display typical imaging features of MS. Tumor size varied from 2 mm to 140 mm with a mean value of 37.5 ± 25.3 mm.

There were no statistically significant differences for tumor size in regard of the hematological disease (for AML mean value 37.9 ± 26.6 mm, for CML 32.4 ± 18.7, for secondary AML 42.7 ± 22.7 mm, *p* = 0.28).

There was no difference in tumor size between incidental lesions and symptomatic lesions (33.8 ± 33.6 mm versus 38.8 ± 25.2, *p* = 0.41).

### CT

For 57/151 patients (37.7%) CT images were available. Every lesion showed a moderate contrast enhancement after intravenous contrast media application. The contrast enhancement was homogenous in 40/57 patients (70.2%), and inhomogeneous in 17/57 patients (29.8%).

### MRI

For 54/151 patients (35.8%) MRI was available. On T2-weighted images, 7/54 lesions (13.0%) were hypointense, 6/54 lesions (11.1%) were isointense, and 41/54 (75.9%) were hyperintense in comparison to the adjacent muscle tissue.

On T1-weighted images, MS were hypointense in 22/54 cases (40.7%), isointense in 30/54 cases (55.6%) and hyperintense in 2/54 cases (3.7%) compared to the adjacent muscle tissue.

In 52/151 cases (34.4%) intravenous contrast media was applied. Most commonly a moderate homogenous enhancement was shown in 31/52 cases (59.6%). In 20/52 cases (38.5%) an inhomogeneous enhancement was detected. Only 1/52 case (1.9%) showed no enhancement.

### Diffusion weighted imaging (DWI)

Diffusion weighted imaging (DWI) was available for 17/151 patients (11.2%). Different b-values were used according to study center and investigated body region. On ADC maps the signal intensity was in most lower in comparison to the adjacent muscle tissue (*n* = 14/17, 82.4%). Quantified, the mean apparent diffusion coefficient (ADC) value was 0.76 ± 0.19 × 10^− 3^ mm^2^/s, range 0.42–2.4 × 10^− 3^ mm^2^/s.

Finally, 2/151 patients (1.3%) were investigated with FDG-PET. The lesions showed high elevated tracer-uptake.

## Discussion

The present study provides clinical and imaging findings of MS based upon a large patient sample in a multicenter design.

MS most commonly occurs in patients with AML with a reported incidence of 2.5–9% [2, 4]. Furthermore, MS can less commonly occur in other hematological diseases, such as CML, MDS and myeloproliferative diseases with yet no reported systematic data regarding incidences. Concordantly, only few patients with these entities were identified in our patient sample.

Making the diagnosis of MS can be a challenge, especially in patients with extramedullary MS and normal appearing bone marrow as a first manifestation of AML [6]. These patients represent, however, less than 1% of all AML cases [6]. MS is associated with an overall poor survival with reported median survival of 12.8 to 15.9 months [6, 8, 12]. In short, sole presence of MS indicates poor outcome irrespective of the clinical setting.

### Clinical findings

Principally, MS can be classified into 4 groups according to the manifestation. First is the manifestation of MS with concurrent acute myeloid leukaemia. In such cases the diagnosis of MS might be easy to make [5, 12, 13]. Secondly, extramedullary relapse of AML, including in the setting after bone marrow transplantation. Thirdly, blast phase/transformation of a myeloproliferative neoplasm or chronic myelomonocytic leukaemia [5, 12, 13]. Lastly, isolated MS, which occurs in association with a normal bone marrow biopsy and blood analysis, and in the absence of any history of myeloid neoplasia [6]. These cases of MS might be most challenging in diagnosis and needs histopathological evaluation of the MS suspicious lesion [6, 12, 13].

The identified frequencies in the present study are well comparable with the literature. Most common the extramedullary relapse setting of AML with up to 60%, followed by simultaneous manifestation in 30% and less frequently, as an extramedullary manifestation of AML without bone morrow involvement in only few cases.

The diagnosis of MS can be challenging, and relatively high misdiagnosis rates have been reported varied from 25 to 47%, which especially concern de novo manifestations without bone marrow involvement [12–14]. Possible misdiagnoses comprised Hodgkin-Lymphomas, MALT lymphoma, or Ewing’s sarcoma, which can show similar histopathologic characteristics [12].

Clinically, infectious diseases such as abscesses or hematomas should be considered as a possible differential diagnosis because these occur very frequently in leukemic patients with immunosuppression and thrombocytopenia, either due to chemotherapy or due to malignancy itself [8].

Regarding gender, a slight male predominance was identified in the literature [6, 13]. However, in the present sample there was no gender predominance.

Clinical presentation of MS largely depends on the affected site. Correspondingly, MS can present with various symptoms, such as tumor mass effect or local organ dysfunction [2]. However, according to the literature, about half of patients with MS were asymptomatic and, therefore, they were identified by imaging [8]. Notably, in the present study, most cases of MS (24.5%) were detected incidentally by imaging studies, which is caused by increasing usage of cross-sectional imaging in hematological patients. Therefore, the radiologist and oncologist need to be aware of MS.

Regarding localizations, MS affects most frequently cutis, subcutis and lymph nodes [5, 9, 13]. However, the frequencies of different localizations varied in several studies. In fact, Kaur et al. reported a skin involvement in up to 69.5% of patients in a case series of 22 patients [5]. Contrary, Pileri et al. reported a skin manifestation in only 28.2% of patients based upon 74 patients, which was yet the most common localization [13]. Kawamoto et al. reported clinicopathological findings of 131 patients with MS and identified that lymph nodes were the most frequent manifestation (55%) [3]. Recently, a frequent involvement of the visceral soft tissue (29.9%) as the most common localization was described [9]. MS can principally manifest in every organ resulting in rare organ manifestations including pulmonary, kidney, vaginal and uterine [8]. In the present study, most commonly cutis involvement was found, followed by bone and lymphatic tissue.

A relatively high amount of breast manifestations was identified in our patient sample. Contrary, in previous patient samples no breast manifestation was reported. This might be caused by a university hospital included in the study with a large breast center and therefore possible selection bias.

The affection of the central nervous system is rare with a reported frequency of 1.5%, which can have a crucial impact on the clinical course of patients due to early neurological deficits [3]. We identified a higher rate, namely 9.3% of all acquired cases. Presumably, this might be caused by an increasing use of cross-sectional imaging in oncologic patients, which leads to more incidentally detected lesions. This might explain differences to older case series with lower reported frequency [15].

### Imaging findings

Previously, only few reports with relatively small numbers of patients/lesions reported imaging findings in MS [9–11, 16, 17]. So far, Shinagare et al. described MRI features of 25 patients with 41 different MS localizations [16]. The authors reported that the lesions had a mean size of 5.6 cm (range 1–20 cm), which is slightly higher than in our observation. Presumably, the results differ slightly due to the fact that in the mentioned study only lesions with MRI were included, whereas in the present study mostly CT was used. A MS lesion investigated by MRI might be a clinical symptomatic lesion with consecutive a larger size, whereas CT more commonly detects incidental lesions, which might be smaller in size.

CT findings of MS were reported to be variable, depending on the site of involvement [8]. Most commonly, on CT images, MS lesions were reported to be isodense to adjacent muscle tissue [9]. In contrast, cerebral manifestations were reported to be slightly hyperdense compared to the bordering brain [17]. After application of contrast medium, in most cases a homogenous enhancement was observed [9]. The homogenous CT-texture might reflect the histopathology and help to distinguish other malignant tumors with more necrotic areas. However, studies are needed to employ this imaging feature for discrimination purposes.

The present study corroborates the previous results with overall good comparable frequencies in regard of contrast media characteristics and density of CT images.

Regarding MRI, it was reported that 75.6% of MS lesions were isointense and 24.4% were hypointense on T1-weighted images [16]. On T2-weighted images, 95.1% were hyperintense and 4.9% were isointense [16]. In a recent study based on 28 patients, on T2-weighted images most cases (82.1%) were hyperintense, whereas on T1-weighted images 60.7% of the identified cases were isointense compared to adjacent muscle tissue [9]. Similar results were also reported in another case series [17].

Our results based on overall 54 lesions suggested similar frequencies in regard of signal intensities.

A widely used imaging technique is DWI, which can quantify proton movement in tissues and is, therefore, able to reflect microstructure of tissues [18]. It was previously reported that DWI is very sensitive for lymphoma lesions, which show a comparable histopathology characteristic as MS lesions [19]. So, it was reported that most MS lesions showed a diffusion restriction with up to 96% of cerebral lesions [10]. Similar results were identified based upon 10 cases with a more advanced imaging protocol [20]. Moreover, the ADC value increases after therapy [20]. In the present study, the mean ADC value was slightly higher than reported, yet with most a diffusion restriction pattern. This is most likely caused by a high cellularity of the lesions, as it was extensively investigated that ADC values are inversely correlated with tissue cellularity [18]. DWI might be a useful diagnostic tool for treatment evaluation of MS, which needs, however, more data. In regard of differential diagnosis, however, other malignant tumors and abscesses can also show restricted diffusion, which might reduce the diagnostic value of this sequence in clinical routine [21].

A beneficial imaging modality is FDG-PET/CT, which has a better accuracy than CT in diagnosing MS [22–24]. As reported previously, MS lesions show typically an intense FDG uptake. More interestingly, the tracer uptake changed under therapy, which correlated with clinical outcome [22–24]. Moreover, FDG-PET can detect additional lesions, which were not clinically known [24]. Yet, systematic data are still needed for this imaging modality to prove possible benefit. Only few lesions in the present study were staged with PET/CT, which also showed an elevated FDG uptake in good agreement with the literature.

There are several limitations of the present study to address. Firstly, it is a retrospective study with known possible inherent bias. Secondly, the patient sample is recruited from 4 German university hospitals with possible different diagnosis and treatment regimens and imaging studies were not available for all patients. Thus, due to the multicenter design the present patient sample is relatively large. Furthermore, multicenter prospective studies are difficult to perform due to low incidence of MS. Thirdly, the imaging analysis was performed in the involved centers and no central reading was performed, which might harbor some possible differences in regard of imaging assessment. However, the reading was performed by board certified radiologists and can, therefore, be generalized for clinical routine. Fourthly, the diagnosis of MS was not histopathologically confirmed in all cases. It has been reported that the misdiagnosis rate of MS is up to 47% [2], which might be substantial lower in the present patient sample because only clinical highly suspicious MS-lesions were included in the analysis.

The oncologist and radiologist need to keep in mind the diagnosis of MS for every unclear and new occurrent lesion in a patient with known AML, independently from the localization.

## Conclusions

Myeloid sarcoma is a rare manifestation of several hematological malignancies, most common in AML, which can affect any part of the body. Most commonly, it manifests within the skin and bone, followed by lymphatic tissue. This multicenter retrospective study provides clinical and imaging findings based upon a large patient sample.

## Data Availability

The datasets used and/or analysed during the current study available from the corresponding author on reasonable request.

## Abbreviations

ADC: Apparent diffusion coefficient
AML: Acute myeloid leukemia
CML: Chronic myeloid leukemia
CMML: Chronic myelomonocytic leukemia
CNS: Central nervous system
CT: Computed tomography
DWI: Diffusion weighted imaging
FDG-PET: Fluorodeoxyglucose-Positron-emission tomography
MDS: Myeloid dysplastic syndrome
MPS: Myeloid proliferative syndrome
MRI: Magnetic resonance imaging
MS: Myeloid sarcoma
